# Effects of stepwise administration of osteoprotegerin and parathyroid hormone-related peptide DNA vectors on bone formation in ovariectomized rat model

**DOI:** 10.1038/s41598-024-51957-0

**Published:** 2024-01-30

**Authors:** Ye Ji Eom, Jang-Woon Kim, Yeri Alice Rim, Jooyoung Lim, Se In Jung, Ji Hyeon Ju

**Affiliations:** 1 Catholic iPSC Research Center (CiRC), CiSTEM Laboratory, College of Medicine, The Catholic University of Korea, Seoul, Republic of Korea; 2 Department of Biomedicine and Health Science, College of Medicine, The Catholic University of Korea, Seoul, Republic of Korea; 3 Division of Rheumatology, Department of Internal Medicine, Institute of Medical Science, College of Medicine, Seoul St. Mary’s Hospital, The Catholic University of Korea, Seoul, Republic of Korea

**Keywords:** Biological techniques, Diseases

## Abstract

Osteoporosis is a metabolic bone disease that impairs bone mineral density, microarchitecture, and strength. It requires continuous management, and further research into new treatment options is necessary. Osteoprotegerin (OPG) inhibits bone resorption and osteoclast activity. The objective of this study was to investigate the effects of stepwise administration of OPG-encoded minicircles (mcOPG) and a bone formation regulator, parathyroid hormone-related peptide (PTHrP)-encoded minicircles (mcPTHrP) in osteoporosis. The combined treatment with mcOPG and mcPTHrP significantly increased osteogenic marker expression in osteoblast differentiation compared with the single treatment groups. A model of postmenopausal osteoporosis was established in 12-week-old female rats through ovariectomy (OVX). After 8 weeks of OVX, mcOPG (80 µg/kg) was administered via intravenous injection. After 16 weeks of OVX, mcPTHrP (80 µg/kg) was injected once a week for 3 weeks. The bone microstructure in the femur was evaluated 24 weeks after OVX using micro-CT. In a proof-of-concept study, stepwise treatment with mcOPG and mcPTHrP on an OVX rat model significantly improved bone microstructure compared to treatment with mcOPG or mcPTHrP alone. These results suggest that stepwise treatment with mcOPG and mcPTHrP may be a potential treatment for osteoporosis.

## Introduction

Osteoporosis is a metabolic bone disease that affects bone mineral density (BMD), bone mass, microarchitecture, and strength, leading to increased bone turnover and fracture risk^1^. There are two categories of osteoporosis: primary and secondary. Primary osteoporosis is the most common form and occurs due to natural aging with no association with other chronic diseases. Primary osteoporosis is classified into two types: type 1 postmenopausal osteoporosis and type 2 senile osteoporosis. Furthermore, primary osteoporosis can be caused by factors such as calcium or vitamin D deficiency, smoking, and alcohol consumption^2^. In contrast, secondary osteoporosis is caused by chronic medical conditions or medication use. Secondary osteoporosis can be caused by chronic diseases such as endocrine, gastrointestinal, and autoimmune diseases, as well as medications and other factors^3^.

Because osteoporosis can be caused by menopause, women are more at risk than men^4^. Menopause-induced estrogen deficiency disrupts normal bone replacement cycle, increases osteoclast resorption, and decreases osteoblast activity. Moreover, an imbalance between the osteoclasts and osteoblasts causes bone loss and increases fracture risk^5,6^. Osteoclasts are located on the bone surface and play an important role in resorption. Receptor activator of nuclear factor-κB ligand (RANKL) induces osteoclast differentiation by binding to RANK, and activated osteoclasts secrete acids and proteolytic enzymes that induce bone loss^7^. Bone loss in postmenopausal women occurs in two stages. In the early stage, bone resorption increases disproportionately with bone formation, causing a rapid loss of cancellous bone, whereas there is a sustained and slow loss of cancellous and cortical bones in the second stage. Besides decreased trabecular bone thickness and connectivity, the bone plate changes into a rod-like structure in postmenopausal women, which increases the risk of osteoporotic fractures^8,9^.

Medications for postmenopausal osteoporosis are classified as antiresorptive, anabolic, or dual mechanism agents^10–12^. Antiresorptive agents reduce fracture risk by suppressing osteoclast-mediated bone resorption and increasing BMD^13^. Examples of antiresorptive agents include bisphosphonates, selective estrogen receptor modulators (SERMs), calcitonin, and monoclonal antibodies against RANKL, such as denosumab. Anabolic drugs, such as teriparatide and abaloparatide, promote new bone formation by osteoblasts and increase BMD. Anabolic drugs, such as teriparatide and abaloparatide, promote new bone formation by osteoblasts and increase BMD. They are used to reduce the risk of fractures in high-risk patients^14^. Anabolic drugs, such as teriparatide and abaloparatide, promote new bone formation by osteoblasts and increase BMD. Romosozumab is a pharmacological treatment with a dual mechanism of action, both anabolic and antiresorptive, that increases bone strength by decreasing bone turnover and restoring bone mineral content in osteoporosis^15^.

As our understanding of the fundamental mechanisms that cause osteoporosis has improved, treatments for the condition have advanced through the development of various drugs. The diversity of osteoporosis treatment options has expanded, leading to more choices in drug selection and treatment order. The treatment of osteoporosis necessitates the development and implementation of a personalized treatment plan based on various factors, including the patient's fracture status, medication adherence, and response to drug therapy^16,17^. Multiple scientific organizations have released statements and guidelines outlining the sequential treatment of osteoporosis based on the fracture risk of patients with the condition^14,16,18^. Various sequential treatment options using different agents are being studied for the management of osteoporosis, such as antiresorptive agents followed by anabolic agents, anabolic agents followed by antiresorptive agents, and antiresorptive agents followed by antiresorptive agents^19–21^. However, as there is currently no complete cure for osteoporosis, further research is necessary to develop and explore new treatment options.

Parathyroid hormone-related peptide (PTHrP) is a protein homologous to parathyroid hormone (PTH) at the amino terminus that binds to the same G-protein-coupled receptor^22^. Teriparatide (PTH 1–34), a fragment of recombinant human PTH, is critical for bone formation and increases bone mass and strength^23^. For example, PTH 1–34 treatment reduces the risk of vertebral and nonvertebral fractures and increases vertebral, femoral, and systemic BMD in postmenopausal osteoporosis^24,25^. Abaloparatide, a synthetic PTHrP analog with an amino acid substitution between positions 22 and 31 of PTHrP (1–34), shares 78% sequence homology with teriparatide, is an anabolic agent for postmenopausal osteoporosis^26–29^. Treatment with these anabolic agents is recommended for postmenopausal osteoporosis cases with high risk of fracture and PTHrP overexpression has been reported to improve bone formation in ovariectomized (OVX) mice^17,30^.

Another agent that may be important in osteoporosis treatment is osteoprotegerin (OPG). OPG is a decoy receptor for RANKL expressed in osteoblasts and several tissues, including the heart, kidney, liver, spleen, and bone marrow^31^. In addition, OPG inhibits osteoclast differentiation by blocking the interaction between RANKL and RANK on the osteoclast surface^32,33^. In osteoporosis, bone resorption by osteoclasts increases due to an imbalance between osteoclast and osteoblast activity. OPG can reduce bone loss by suppressing osteoclast differentiation and activation in osteoporosis.

In this study, we hypothesized that sequential treatment with OPG and PTHrP may improve menopausal osteoporosis by suppressing bone resorption and promoting bone formation. In vivo OPG and PTHrP overexpression were induced by delivering non-viral DNA minicle vectors encoding each gene in osteoporotic rat models. We aimed to examine the effects of stepwise injection of OPG-encoded minicircles (mcOPG), which inhibits osteoclast activity, and PTHrP-encoded minicircles (mcPTHrP), which promotes bone formation on osteoporosis by observing its effect in bone quality and microstructure.

## Results

### Generation of minicircles encoding OPG and PTHrP genes

**Figure 1:**
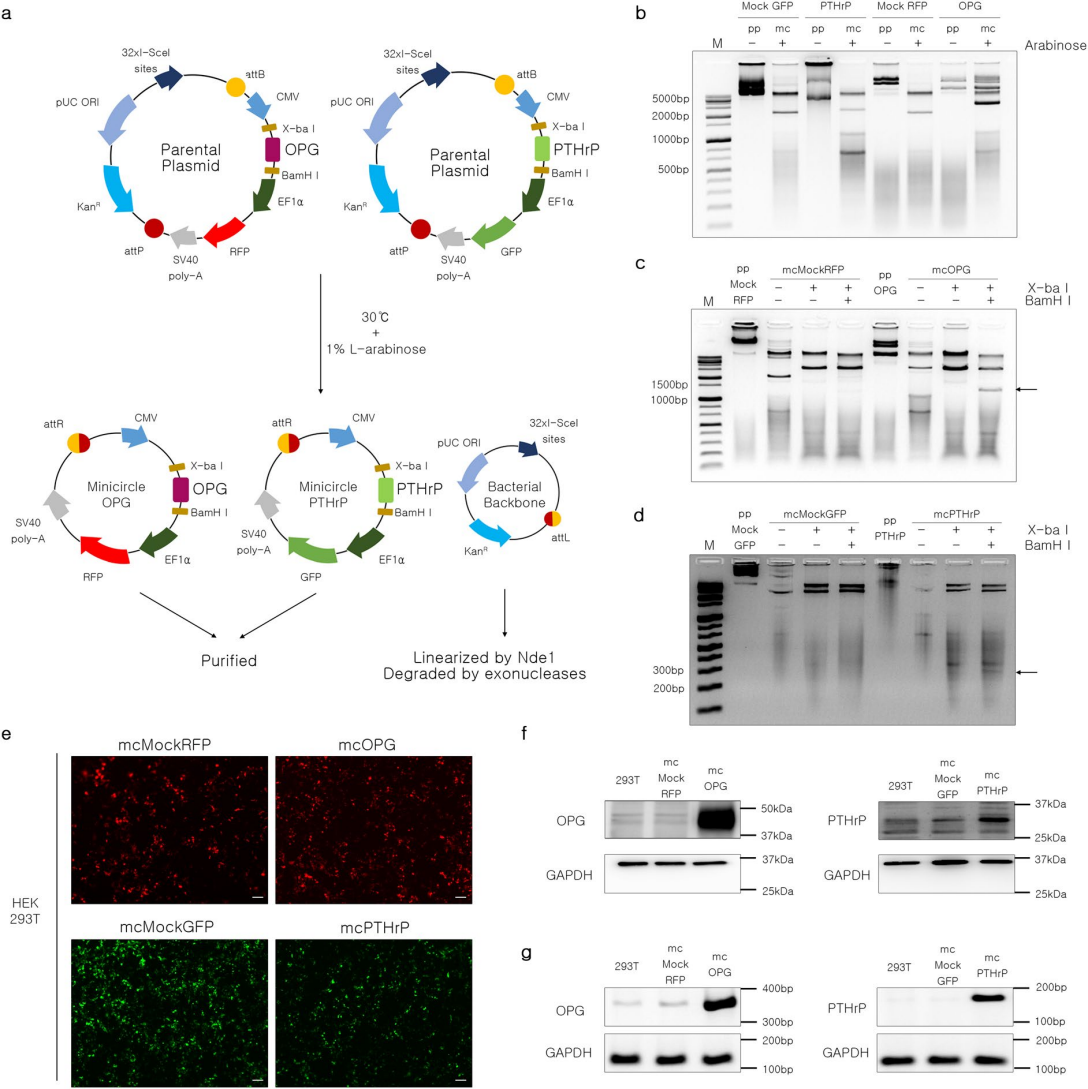
Generation of OPG- and PTHrP-encoding minicircles. (**a**) Design of minicircle encoding OPG and PTHrP. (**b**) Gel electrophoresis of parental plasmid vectors (pp) and minicircle vectors. (**c**,**d**) OPG and PTHrP were cut using the restriction enzymes BamHI and XbaI, respectively. (**e**) Fluorescence microscopy image of transfected HEK293T cells. Scale bar = 100 μm. (**f**) Western blots of mcOPG and mcPTHrP protein expression in HEK293T cells transfected with minicircles. (**g**) Gel image of mcOPG and mcPTHrP mRNA expression in transfected HEK293T cells. *ppMock* parental mock vector, *mcMock* minicircle mock vector, *ppOPG* parental osteoprotegerin–encoding vector, *mcOPG* minicircle encoding osteoprotegerin, *ppPTHrP* parental parathyroid hormone-related protein–encoding vector, *mcPTHrP* minicircle encoding parathyroid hormone-related protein.

Parental plasmids encoding OPG and PTHrP were designed and synthesized (Fig. 1a). The plasmids were divided into minicircles and bacterial backbones by inducing recombination using arabinose (Fig. 1b). Successful insertion of the OPG (1,205 bp) and PTHrP (282 bp) sequences was confirmed by double digestion with XbaI and BamHI (Fig. 1c and d). HEK293T cells were transfected with the generated minicircles. The expression of red (mcOPG, mcMockRFP) and green (mcPTHrP, mcMockGFP) fluorescent proteins was confirmed 48 h after transfection (Fig. 1e). Transfected HEK293T cells showed higher mRNA and protein expression of OPG and PTHrP compared to non-transfected (control) and mcMock-transfected cells (Fig. 1f and g). The results confirm the successful cloning of mcOPG and mcPTHrP. The minicircles carrying each DNA sequence efficiently express OPG and PTHrP protein.

### Osteoblast differentiation of mcOPG- and mcPTHrP-transfected hiPSCs

**Figure 2:**
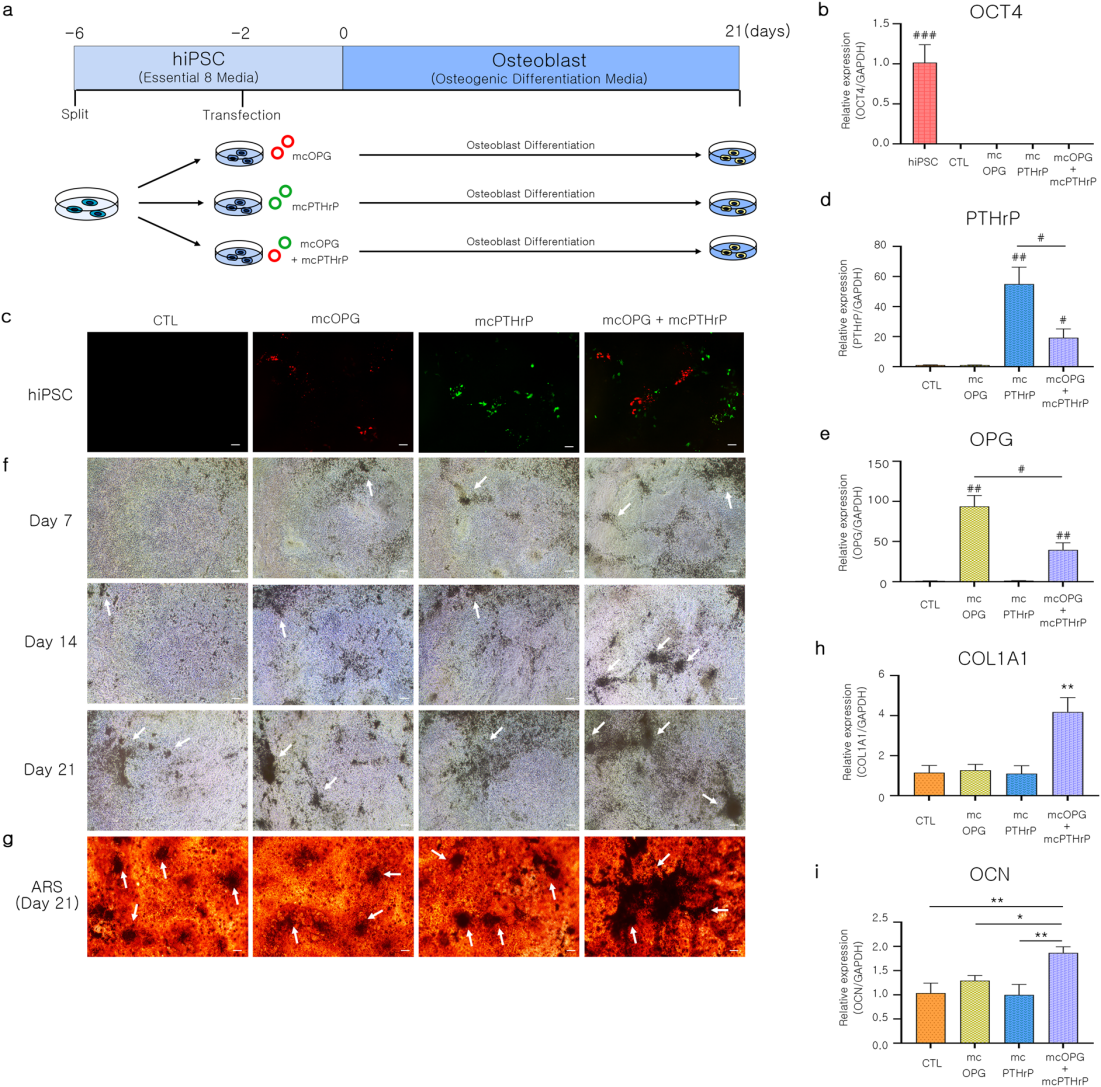
Effects of transfection with OPG- and PTHrP-encoding minicircles on the differentiation of hiPSCs to osteoblast. (**a**) Culture scheme of OPG or PTHrP minicircle-transfected hiPSC-derived osteoblast. (**b**) Relative gene expression of the pluripotent marker OCT4. Data are presented as mean ± SEM. Welch's ANOVA test was performed, and t-test was applied to analyze comparisons between groups; ^#^*P* < 0.05; ^##^*P* < 0.01; ^###^*P* < 0.001. (**c**) Fluorescence microscopy image of transfected hiPSCs. GFP represents the expression of PTHrP, and RFP represents the expression of OPG. Scale bar = 100 μm. (**d**,**e**) Relative gene expression of (**d**) PTHrP and (**e**) OPG genes in hiPSCs 48 h after transfection with the minicircles. Data are presented as mean ± SEM. Welch's ANOVA test was performed, and t-test was applied to analyze comparisons between groups; ^#^*P* < 0.05; ^##^*P* < 0.01; ^###^*P* < 0.001. (**f**) Microscope images at 7, 14, and 21 days after the induction of osteoblast differentiation. The white arrows indicated mineralized nodule formation. Scale bar = represents 100 μm. (**g**) ARS staining image staining mineralized nodule formation of osteoblast 21 days after inducing osteoblast differentiation. The white arrow indicated mineralized nodule formation. Scale bar = represents 100 μm. (**h**,**i**) Relative gene expression of the osteoblast differentiation-related marker genes (**h**) COL1A1 and (**i**) OCN in osteoblasts 21 days after differentiation induction. Data are presented as mean ± SEM. One-way ANOVA; ^*^P < 0.05; ^**^P < 0.01; ^***^P < 0.001, LSD test. *mcOPG* minicircle osteoprotegerin–encoding vector, *mcPTHrP* minicircle parathyroid hormone-related protein–encoding vector, *GFP* green fluorescent protein, *RFP* red fluorescent protein, *OCT4* octamer-binding transcription factor 4, *ARS* alizarin red s, *COL1A1* collagen type I alpha 1 chain, *OCN* osteocalcin, *OPG* osteoprotegerin.

To confirm the effects of mcOPG and mcPTHrP on in vitro osteogenesis, osteoblasts were differentiated from hiPSCs transfected with mcOPG, mcPTHrP, and mcOPG + mcPTHrP minicircles. The hiPSCs were transfected with the minicircles two days before osteoblast differentiation, and the cells were differentiated for 21 days (Fig. 2a). The relative expression of the pluripotency marker gene OCT4 was significantly decreased in the differentiated osteoblasts compared to its levels in hiPSCs (Fig. 2b). Furthermore, the expression of red and green fluorescent proteins (RFP) and GFP was verified in hiPSCs after transfection with the minicircles. The results were comparable to those observed in transfected HEK293T cells (Fig. 2c). The mRNA expression of PTHrP was higher in mcPTHrP and mcOPG + mcPTHrP-transfected hiPSCs than in non-transfected (control) and mcOPG transfected cells. (Fig. 2d) The mRNA expression level of OPG was higher in mcOPG and mcOPG + mcPTHrP-transfected hiPSCs compared with non-transfected and mcPTHrP-transfected cells (Fig. 2e). During in vitro culture, differentiated osteoblasts form mineralized nodules^34,35^, which were observed as brown and dark-colored nodules (indicated by white arrows) under the microscope on days 7, 14, and 21 of osteoblast differentiation (Fig. 2f). Mineralized nodule formation was most abundant on day 21, as confirmed by staining osteoblasts with alizarin red S (ARS), which specifically stains calcium deposits within mineralized nodules formed during osteogenic culture^36^. Mineralized nodules were observed in all groups (indicated by white arrows), with more nodules formed in the mcOPG + mcPTHrP group compared to other groups (Fig. 2g). Osteoblasts secrete and mineralize the extracellular matrix, which is primarily composed of collagen type I and contains smaller but significant amounts of other proteins, such as osteocalcin (OCN). OCN has been shown to promote mineral deposition and increases during matrix mineralization^37^. To assess osteoblast differentiation markers, we analyzed the relative gene expression of collagen type I (COL1A1) and OCN (Fig. 2h and i). The expression levels of osteocalcin (OCN) and collagen type I alpha 1 (COL1A1) were significantly higher in the group overexpressing both macrophage colony-stimulating factor (mcOPG) and parathyroid hormone-related protein (mcPTHrP) compared to the control, mcOPG, and mcPTHrP groups. These findings suggest that overexpression of OPG and PTHrP enhances osteoblast differentiation and maturation in vitro.

### In vivo delivery and expression confirmation of mcOPG and mcPTHrP

**Figure 3:**
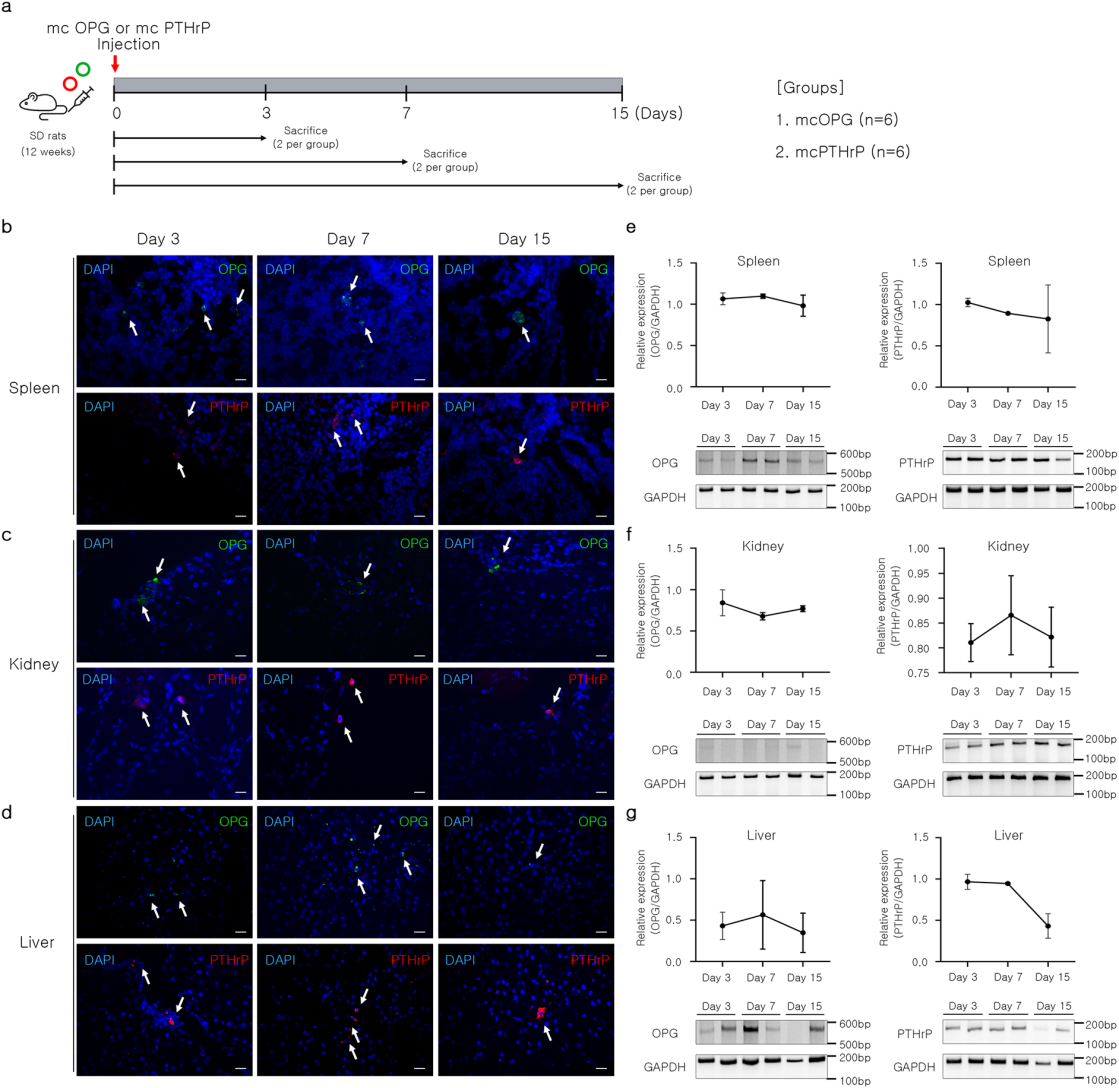
Expression of OPG and PTHrP-encoding minicircles in vivo. (**a**) Scheme of the injection of minicircles encoding OPG and PTHrP to rats via tail vein. (**b**–**d**) Immunofluorescence images of (**b**) spleen, (**c**) kidney, and (**d**) liver stained with OPG and PTHrP antibodies at 3, 7, and 15 days after intravenous (IV) injection with mcOPG and mcPTHrP via tail vein. Scale bar = 100 μm. (**e**–**g**) Relative expression of the OPG and PTHrP gene in (**e**) spleen, (**f**) kidney, and (**g**) liver. The two bands at each time point shown in the gel image represent the two rats analyzed at each time point. Results are presented as the mean band intensity normalized to that of GAPDH (Image J software). *mcOPG* minicircle encoding osteoprotegerin, *mcPTHrP* minicircle encoding parathyroid hormone-related protein, *OPG* osteoprotegerin, *PTHrP* parathyroid hormone-related protein.

Previous studies have demonstrated that the generated minicircles promote in vitro osteogenesis. The highest differentiation efficacy was observed with the overexpression of both vectors. Before injecting the minicircles into disease model rats, we confirmed their efficacy in vivo using normal rats. To determine protein expression derived from mcOPG and mcPTHrP delivered to the kidney, spleen, and liver, two rats from each group were sacrificed on days 3, 7, and 15 after injection (Fig. 3a). Immunofluorescence staining confirmed OPG and PTHrP protein expression in rat tissues following minicircle injection (Fig. 3b–d, indicated by white arrows), as evidenced by GFP and RFP expression in each tissue. The successful in vivo delivery of mcPTHrP and mcOPG in the tissues of two individual rats at 3, 7, and 15 days after injection was confirmed by the increased gene expression of OPG and PTHrP (Fig. 3e–g). Both mcOPG and mcPTHrP were expressed in the spleen (Fig. 3e). The expression of OPG was relatively low in the kidney compared to the other tissues (Fig. 3f). Figure 3g reveals the liver's minicircles demonstrating a comparable trend to the spleen. These findings demonstrate the successful in vivo delivery of mcOPG and mcPTHrP, resulting in the production of OPG and PTHrP.

### Stepwise injection of mcOPG and mcPTHrP improves the bone microarchitecture in OVX

**Figure 4:**
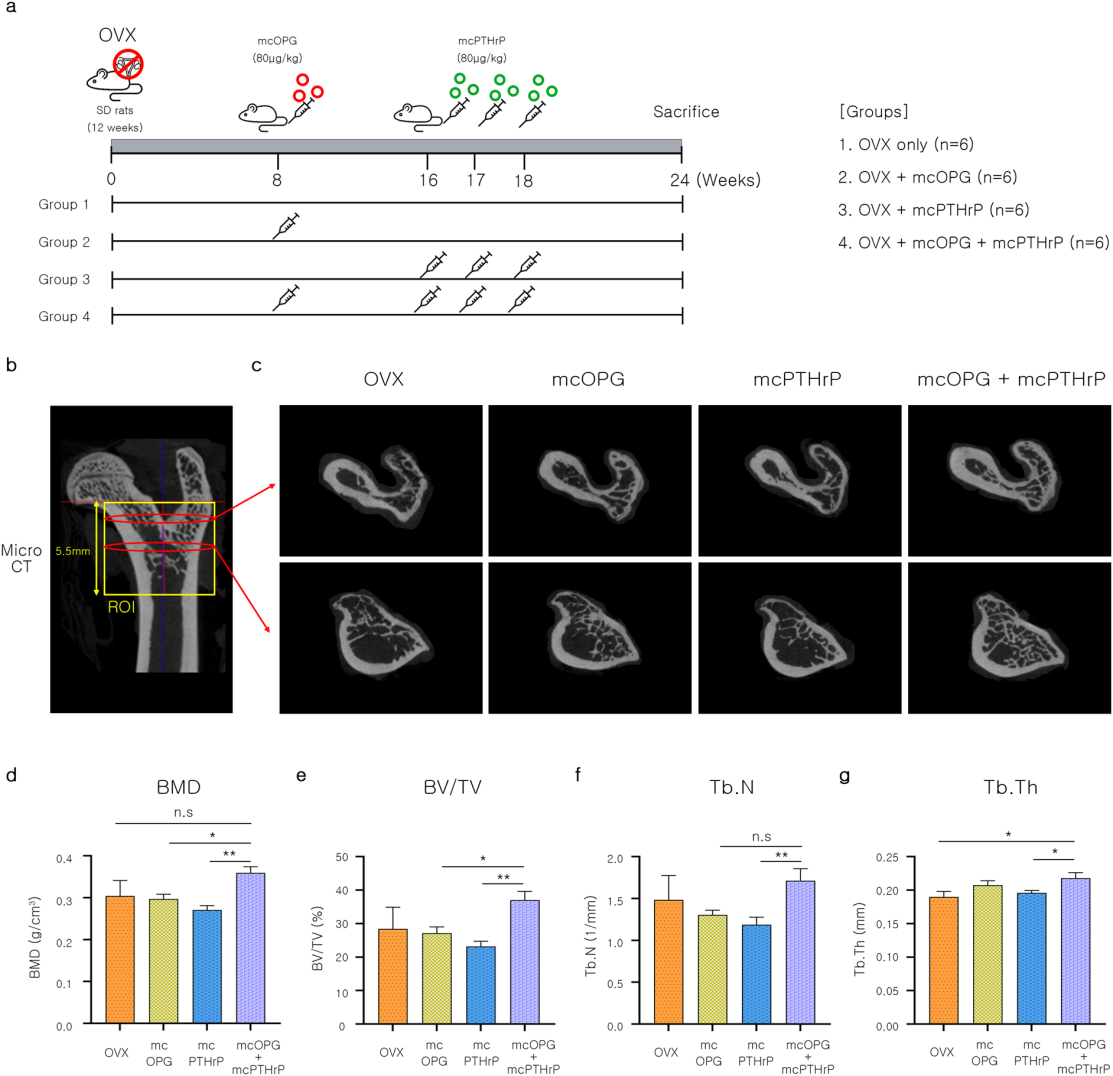
Bone related effect of OPG and PTHrP-encoding minicircles in OVX rats. (**a**) Scheme of the injection of minicircles encoding OPG and PTHrP to OVX rat models via tail vein. (**b**) ROI images for micro-CT analysis. (**c**) Micro-CT images of rat metaphysis femurs from each group. (**d**–**g**) Micro-CT was performed to evaluate bone microstructure-related parameters of the femurs of OVX rats injected with minicircles encoding OPG and PTHrP. (**d**) Bone mineral density (BMD), (**e**) trabecular bone volume fraction (BV/TV), (**f**) trabecular bone number (Tb.N), and (**g**) trabecular bone thickness (Tb.Th). Data are presented as mean ± SEM. One-way ANOVA; ^*^P < 0.05; ^**^P < 0.01; ^***^P < 0.001; *n. s.* not significant, LSD test. *OVX* ovariectomy, *mcOPG* minicircle encoding osteoprotegerin, *mcPTHrP* minicircle encoding parathyroid hormone-related-protein, *OPG* osteoprotegerin, *PTHrP* parathyroid hormone-related protein, *ROI* region of interest.

To confirm the effect of minicircles on in vivo bone regeneration, we generated osteoporosis rat models via OVX. After 8 weeks, mcOPG (80 μg/kg) was injected, followed by mcPTHrP (80 μg/kg) after an additional 8 weeks (Fig. 4a). We compared the stepwise injected group to each single injected group. We collected OVX rat femurs after 24 weeks for bone quality assessment, including BMD. A region of interest (ROI) was designated for assessing trabecular bone in the femur (Fig. 4b). Micro-CT images revealed that the microstructure of trabecular bone was improved with stepwise injections of mcOPG and mcPTHrP, compared to the other groups (see Fig. 4c). Furthermore, stepwise injection of mcOPG and mcPTHrP significantly increased BMD compared to the other groups (Fig. 4d). The stepwise injected group had a significantly higher bone volume fraction (BV/TV) compared to the mcOPG and mcPTHrP injected groups (Fig. 4e). Trabecular number (Tb.N) only showed significance between the mcPTHrP injected group and the stepwise injected group (Fig. 4f). All groups showed similar levels in trabecular thickness (Tb.Th); however, the stepwise injected group showed a significant difference between the OVX control group and the mcPTHrP injected group (Fig. 4g). In summary, the mcOPG + mcPTHrP group showed significantly higher BMD, BV/TV, and Tb.N compared to the single injection groups (Fig. 4d–f). Furthermore, the mcOPG + mcPTHrP group had significantly higher Tb.Th compared to the mcPTHrP only group (Fig. 4g). Moreover, supplementary material containing additional parameters related to trabecular bone microstructure, such as bone surface (BS), total volume (TV), bone volume (BV), bone surface density (BS/TV), specific bone surface (BS/BV), trabecular separation (Tb.Sp), degree of anisotropy (DA), connectivity density (Conn.D), and structure model index (SMI), is provided (Supplementary Fig. 5). In addition, histological analysis of the bones was conducted and presented through hemoxylin and eosin (H&E) staining (Supplementary Fig. 5). The results indicate that the stepwise injection of mcOPG and mcPTHrP enhances the quality and microstructure of trabecular bone in OVX rats.

### Stepwise injection of mcOPG and mcPTHrP increase bone formation and inhibits bone resorption in OVX

**Figure 5:**
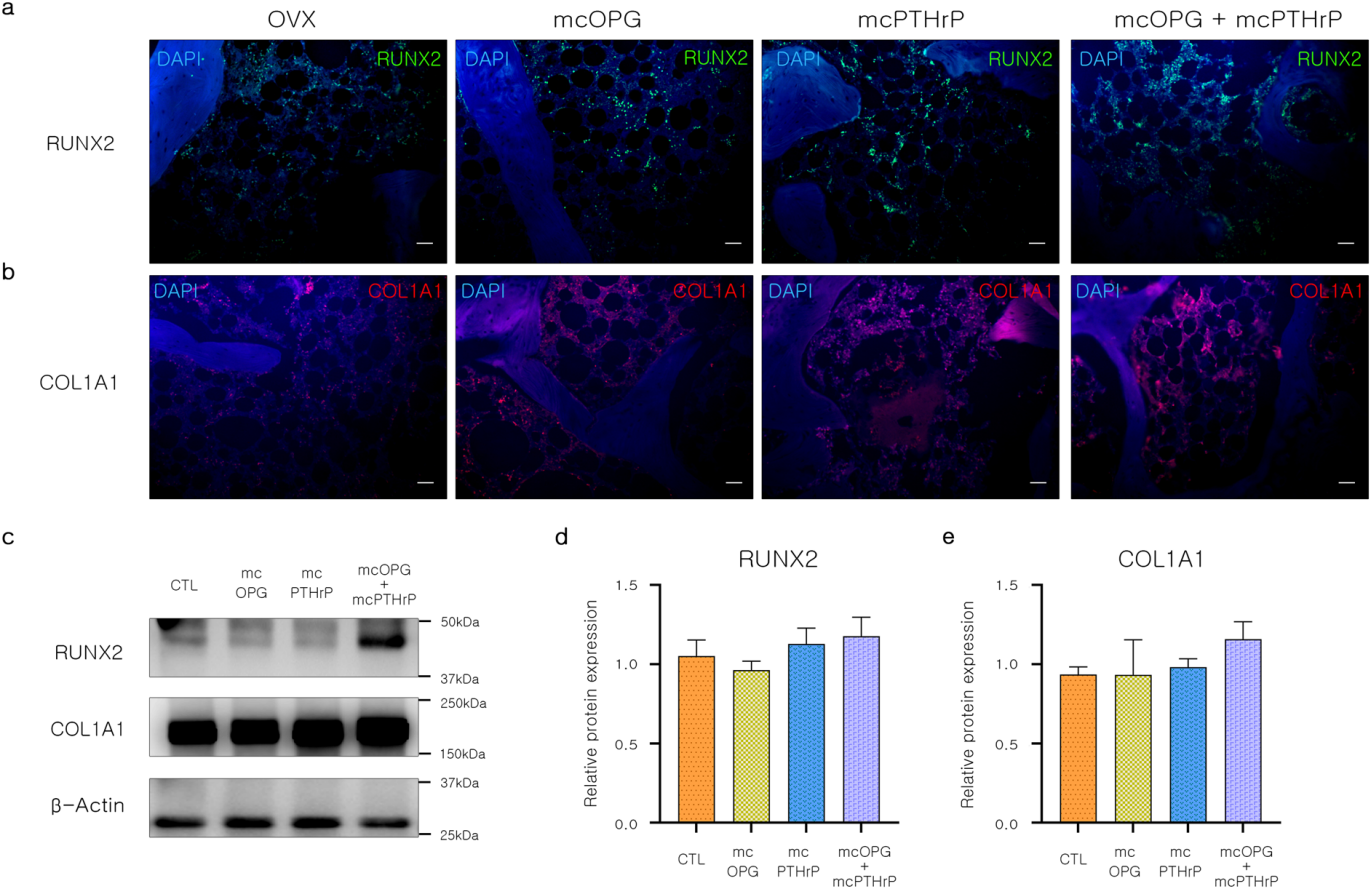
Bone formation related effect of OPG and PTHrP-encoding minicircles in OVX rats. (**a**,**b**) Immunofluorescence images of femur stained with (**a**) RUNX2 and (**b**) COL1A1 antibodies. Scale bar = 100 μm. (**c**) Representative band image. Protein expression of (**d**) RUNX2 and (**e**) COL1A1. Data are presented as mean ± SEM. One-way ANOVA; ^*^P < 0.05; ^**^P < 0.01; ^***^P < 0.001, LSD test. *OVX* ovariectomy, *mcOPG* minicircle encoding osteoprotegerin, *mcPTHrP* minicircle encoding parathyroid hormone-related protein, *OPG* osteoprotegerin, *PTHrP* parathyroid hormone-related protein, *RUNX2* runt-related transcription factor 2, *COL1A1* collagen type I alpha 1 chain 1.

The bone microstructure of OVX rats injected with both mcOPG and mcPTHrP was improved. To investigate the effects of the minicircles, we analyzed the expression of pro-osteogenic protein markers in the femurs of OVX rats injected with the minicircles. Compared to the OVX control group, the mcOPG, mcPTHrP, and mcOPG + mcPTHrP injected groups showed strong expression of RUNX2 (Fig. 5a). Collagen type I (COL1A1) expression levels exhibited a similar trend to that of RUNX2 in the mcOPG, mcPTHrP, and mcOPG + mcPTHrP injected groups (Fig. 5a and b). However, the protein expression of RUNX2 and collagen type I in the mcOPG + mcPTHrP group was not significant (Fig. 5c–e).

**Figure 6:**
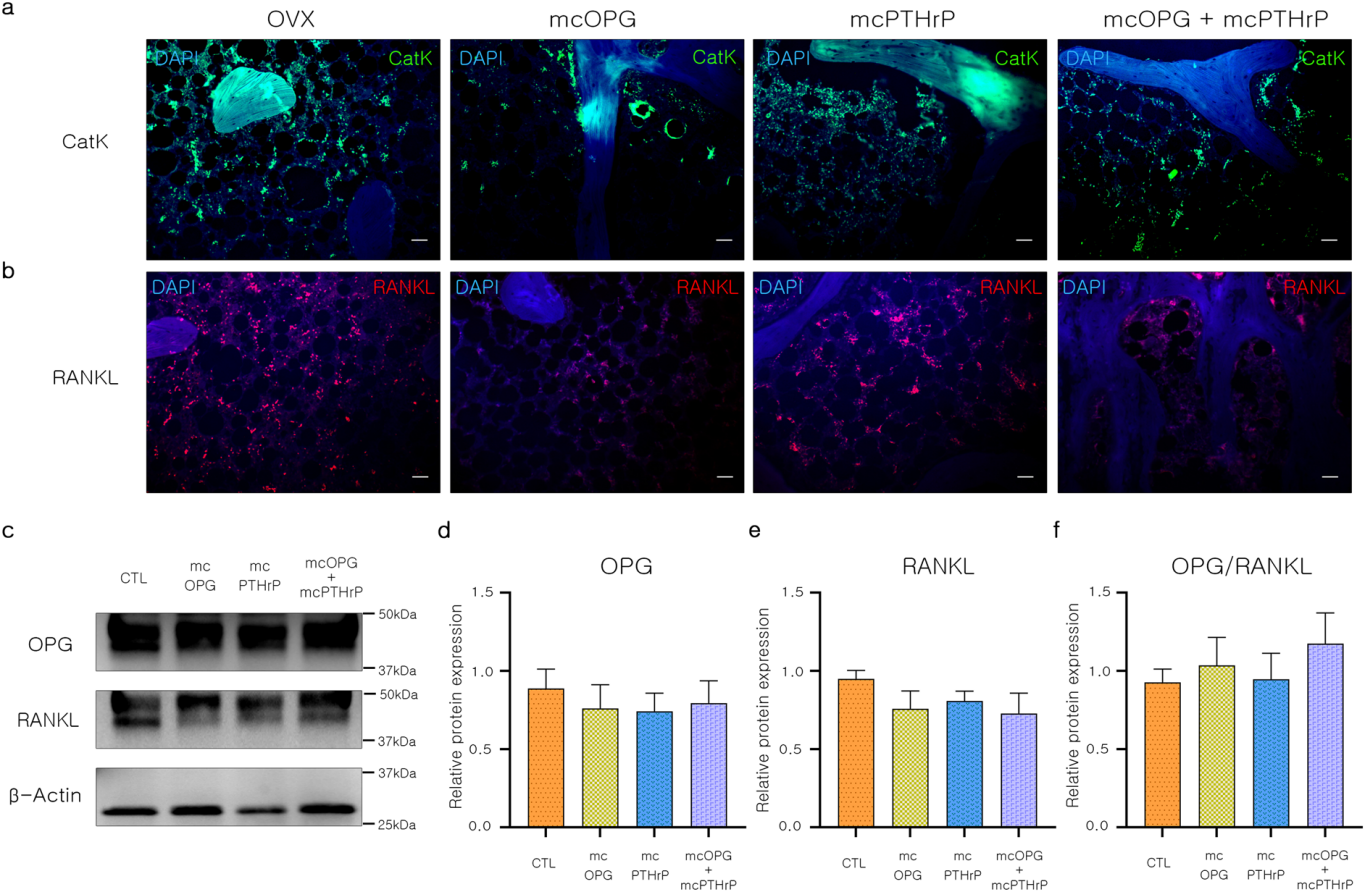
Bone resorption-related effect of OPG- and PTHrP-encoding minicircles in OVX rats. (**a**,**b**) Immunofluorescence images of femur stained with (**a**) CatK and (**b**) RANKL antibodies. Scale bar = 100 μm. (c-f) Protein expression of bone resorption-related markers. (c) Representative band image. (**d**–**f**) protein expression of (d) OPG, (e) RANKL, (f) OPG/PTHrP ratio. Data are presented as mean ± SEM. One-way ANOVA; ^*^P < 0.05; ^**^P < 0.01; ^***^P < 0.001, LSD test. *mcOPG* minicircle encoding osteoprotegerin, *mcPTHrP* minicircle encoding parathyroid hormone-related protein, *OPG* osteoprotegerin, *PTHrP* parathyroid hormone-related protein, *CatK* cathepsin K, *RANKL* receptor activator of nuclear factors κB ligand.

To confirm the effect of the minicircles on bone resorption, we conducted further experiments after inducing OPG expression in OVX rats, which is expected to block osteoclast differentiation. Cathepsin K (CatK) is mainly responsible for the degradation of collagen type I in osteoclast-mediated bone resorption and is predominantly secreted by activated osteoclasts^38,39^. Interestingly, the expression of CatK and RANKL decreased in the mcOPG and mcOPG + mcPTHrP groups compared to the OVX and mcPTHrP groups. The mcOPG + mcPTHrP group had the lowest expression level of CatK (Fig. 6a) and a similar tendency was observed for RANKL (Fig. 6b). Furthermore, western blotting analysis revealed a slight decrease in bone resorption-related markers in the mcOPG and mcOPG + mcPTHrP groups (Fig. 6c–f). Moreover, the expression of OPG and RANKL proteins was lower in the mcOPG, mcPTHrP, and mcOPG + mcPTHrP groups compared to the OVX group (Fig. 6d and e). The ratio of OPG to RANKL is crucial for osteoclast activity in the bone environment, with a higher ratio indicating lower differentiation and maturation of osteoclasts^33^. Although the OPG/RANKL ratio was higher in the mcOPG + mcPTHrP group than in the other groups, the difference was not statistically significant (Fig. 6f). The results suggest that osteoclast activity is more effectively inhibited by stepwise injection of mcOPG and mcPTHrP compared to a single injection. Furthermore, OPG expression by mcOPG may have a stronger impact on osteoclast inhibition.

## Discussion

Osteoporosis is a chronic disease with no cure that can completely reverse its impact. Currently, osteoporosis treatments include bisphosphonates, SERMs, denosumab, teriparatide, and romosozumab. Although these drugs are effective, their side effects limit their duration of use^40^. Therefore, research is needed to develop new drugs and strategies that use existing drugs and techniques more effectively. Various treatment options for osteoporosis and their effects are under study. Studies suggest that administering multiple drugs in combination may be more effective than using a single drug^12,41,42^. In this study, we examined the effect of stepwise treatment of OPG and PTHrP by injecting minicircle vectors, encoding OPG and PTHrP on bone regeneration in OVX rats.

Non-viral vector gene delivery is a promising and safe in vivo gene transfer strategy^43^. Plasmid DNA vectors can be effective; however, the remaining bacterial sequences may induce immune responses by generating antibodies against bacterial proteins. Minicircles are vectors that have had the bacterial backbone and transcription units, including antibiotic resistance genes, removed. Their relatively smaller size promotes the expression of foreign genes both in vitro and in vivo, making them potentially useful in preclinical gene therapy^44,45^. Minicircles were delivered in vivo via hydrodynamic tail vein injections to induce gene expression, which is a safe and efficient method for gene delivery^46^. Previous studies have confirmed the effectiveness of minicircle vectors encoding human proteins^47–50^. In this study, we developed minicircle vectors encoding OPG and PTHrP (Fig. 1). The transfected cells successfully induced self-production of OPG and PTHrP (Fig. 1e–g).

Several studies have investigated the effects of PTHrP on osteoblast differentiation, growth, and survival^51,52^. Furthermore, OPG participates in osteoclast differentiation, and its role in osteoblast differentiation remains controversial^53,54^. As osteoblast differentiation progresses, osteoblasts form more mineralized nodules^55^. The study confirmed an increase in mineralized nodule formation in osteoblasts differentiated from hiPSCs co-transfected with mcOPG and mcPTHrP (Fig. 2g). Moreover, the nodule formation of cells transfected with both minicircles was even greater than mcPTHrP alone. In addition, the cells co-transfected with both minicircles showed a high expression of COL1A1 and OCN, which are pro-osteogenic markers (Fig. 2h and i). The study confirmed the possibility that co-expression of OPG and PTHrP could improve osteogenesis, considering the degree of mineralization nodule formation and the expression of osteogenic markers.

This study suggested that OPG may have other biological roles in improving osteogenic differentiation and bone formation beyond its anti-osteoclastogenic role. OPG may have an anti-apoptotic role in reducing undesired cell death during bone generation within scaffolds, which could also protect newly synthesized mineral deposits^56^. In addition, OPG may recruit mesenchymal stem cells (MSCs) in synergy with bone morphogenetic protein-2 to enhance bone formation^57^. In a previous study conducted by Li and colleagues^56^, it was found that cell priming with OPG treatment during expansion culture increased the osteogenic differentiation ability of MSCs. Continuous treatment of OPG during both MSC expansion and osteogenic differentiation induced osteogenesis to a lesser extent compared to the OPG treatment used only in either cell expansion or differentiation culture. The authors discussed the potential for OPG to induce both anabolic and catabolic effects depending on the cell status. Furthermore, OPG is likely to prime undifferentiated human MSCs for enhanced osteogenesis. However, the mechanism by which co-expression of OPG and PTHrP affects osteoblast differentiation has not yet been revealed in this study. Based on the study by Li et al., NF-kB may be a possible candidate for increasing osteogenic differentiation. Confirming this intracellular molecule in future studies would be beneficial. Moreover, it would be interesting to confirm the effect of co-expressed OPG and PTHrP in osteoclasts or osteoblasts and osteoclast co-culture platforms to further confirm the synergetic effect of OPG and PTHrP in osteoblast and osteoclast activities and crosstalk.

The limitations of this proof-of-concept study must be considered when interpreting the results. The study used OVX modeling on rats to confirm the osteogenic effect of stepwise administration of mcOPG and mcPTHrP to an osteoporosis model. The OVX rat is a commonly used animal model for osteoporosis research due to its low cost and ease of handling. The study mainly focused on female rats as they are a specific model for postmenopausal human osteoporosis^58^. It has been reported that age-related bone loss in male SD rats begins mostly at 9 months of age, after bone growth has completed. Wang et al. suggest that male SD rats can be an appropriate animal model of age-related bone loss in men, as aging male rats showed comparable bone loss. To confirm the therapeutic effect of stepwise treatment of OPG and PTHrP, it is crucial to apply this strategy in male osteoporosis animal models. Furthermore, the small sample size and different number of animals per group limit the evaluation of the therapeutic effects of the suggested strategy. To evaluate the effect of stepwise administration of OPG- and PTHrP-encoding minicircles in vivo, a proof-of-concept study was conducted with a small sample size of 24 animals 6 per group. The minimum number of animals necessary for statistical analysis was used. To ensure humane treatment, rats were euthanized according to humane standards, such as complications due to aging, during the experiment. Some samples were damaged during the sample collection process and were eventually excluded from the project. Therefore, the final number of animals in each group used for analysis was as follows: (1) OVX group (n = 3), (2) OVX + mcOPG group (n = 4), (3) OVX + mcPTHrP group (n = 5), and (4) OVX + mcOPG + mcPTHrP (n = 6). The variation in the number of animals used in the analysis across groups may affect the validity of the statistical analysis. Thus, we intend to give this more consideration and study with a greater number of animals.

Determining the appropriate timepoint and dosage for administering mcPTHrP after mcOPG injection was a critical issue during this study. There are no confirmed reports on the effect of bone formation through stepwise administration in animal experiments, making it difficult to establish set standards. Because bone formation occurs after bone resorption during the bone remodeling process, it is assumed that mcOPG injection could suppress bone resorption and support bone formation. Additional bone formation is then promoted through mcPTHrP. Previous studies suggest that bone resorption occurs over 2 to 3 weeks, whereas bone formation occurs over 4 to 6 months during the bone remodeling process^59,60^. The administration period of mcOPG was determined based on the administration period of denosumab, a resorption inhibitor used to treat osteoporosis. Denosumab is typically administered once every six months in clinical settings^11^. However, we determined that a six-month gap was too long for rat animal testing and instead chose an eight-week gap. This was done to suppress bone resorption and provide a period of bone formation through mcOPG, regardless of age. Based on our previous studies, we selected a weekly injection period of three weeks for mcPTHrP injections, which have shown osteogenic effects^30^. We increased the dose of minicircles based on the body weight of the animals, as our previous research was conducted on mice. In future studies, we aim to confirm the doses and timepoints that can enhance the synergistic effects of mcOPG and mcPTHrP. Furthermore, a comparative analysis between the current group and a new group injected with mcOPG after mcPTHrP could provide further insight into the mechanism.

Although therapeutic options for osteoporosis have increased, no approved therapy has been found to fully restore normal bone integrity in patients^61^. Combination treatment with anabolic and antiresorptive agents has been suggested to improve treatment efficacy. However, attempts to combine teriparatide with bisphosphonates have failed to prove more consistent than monotherapy^62,63^. Recently, a meta-analysis study based on clinical trial publications^64–69^ confirmed the increased efficacy of the combination of teriparatide and denosumab in treating both osteoporosis and postmenopausal osteoporosis.

In conclusion, this proof-of-concept study suggests a potentially new treatment strategy for osteoporosis: the stepwise in vivo delivery of OPG- and PTHrP-encoding minicircles that can induce self-production of protein. Temporarily inducing the expression of OPG and PTHrP in vivo may be a potential gene therapy strategy for osteoporosis. This approach could prevent possible side effects caused by continuous expression while improving bone remodeling. The effect of stepwise injection of mcOPG and mcPTHrP on bone formation in an osteoporosis model was confirmed. The results showed that stepwise injection was more beneficial than a single injection of mcOPG or mcPTHrP. The sequential treatment may have promoted bone formation and inhibited bone resorption, ultimately improving bone quality and microstructure.

## Materials and methods

### Minicircle production

Parental plasmids (OPG and PTHrP, mock RFP, and mock GFP) were purchased from System Biosciences (Palo Alto, CA, USA). The cDNA sequence of codon-optimized human OPG was subcloned into a mock RFP parental plasmid. The cDNA sequence of the codon-optimized human PTHrP (1–34 + 107–139) was subcloned into the mock GFP parent plasmid. These cDNAs were inserted into the XbaI and BamHI restriction sites of the multiple cloning sites downstream of the CMV promoter. Minicircle vectors were generated according to the manufacturer’s instructions. Minicircle DNA vectors were extracted using NucleoBond Xtra plasmid purification kit (Macherey–Nagel, Duren, Nordrhein-Westfalen, Germany). The inserts encoded by the minicircles were confirmed by double digestion with XbaI and BamHI.

### Cell culture

Human embryonic kidney (HEK293T, American Type Culture Collection, Manassas, VA, USA) cells were cultured in Dulbecco’s Modified Eagle Medium (DMEM, Thermo Fisher Scientific, Waltham, MA, USA) supplemented with 7.5% fetal bovine serum (FBS, Thermo Fisher Scientific, Waltham, MA, USA), 1% penicillin/streptomycin (Thermo Fisher Scientific, Waltham, MA, USA), and incubated at 37 °C in a 5% CO_2_ atmosphere. The human induced pluripotent stem cells (hiPSCs) used in this study were generated from umbilical cord blood mononuclear cells (CBMC)^70^. hiPSCs were maintained in vitronectin-coated plates (Thermo Fisher Scientific, Waltham, MA, USA), and media were replaced daily with Essential 8 medium (E8, Thermo Fisher Scientific, Waltham, MA, USA), and incubated at 37 °C in a 10% CO_2_ atmosphere.

### Minicircle transfection

Briefly, HEK293T cells were detached and seeded (3 × ${10}^{5}$) in a 60 mm plate for transfection. The culture medium was replaced with serum and antibiotic-free DMEM one day prior to transfection. Cells were transfected with the minicircle vector using Lipofectamine 2000 (Thermo Fisher Scientific, Waltham, MA, USA), following the manufacturer’s instructions. Successful transfection was confirmed the next day by detecting the expression of RFP and GFP in cells via fluorescence microscopy (Axio Observer. Z1 inverted, Carl Zeiss, Oberkochen, Germany).

hiPSCs were detached and seeded (3 × ${10}^{4}$) in a 12-well plate pre-coated with 0.1% gelatin and serum media for transfection. hiPSCs were cultured in E8 and incubated at 37 °C in a 10% CO_2_ atmosphere for 4 days, followed by transfection with the minicircle vector using the Lipofectamine 3000 reagent (Thermo Fisher Scientific, Waltham, MA, USA), according to the manufacturer’s instructions. Successful transfection was confirmed the next day by detecting RFP and GFP expression in the cells using a fluorescence microscopy.

### Osteoblast differentiation

Briefly, hiPSCs (3 × ${10}^{4}$) were seeded in a 12-well plate pre-coated with 0.1% gelatin and serum media. hiPSCs were cultured in E8 and incubated 37 °C in a 10% CO_2_ atmosphere for 4 days, followed by transfection. After 48 h, E8 medium was replaced with osteogenic differentiation medium (DMEM supplemented with 15% FBS, 100 nM of dexamethasone, 50 μg/mL of ascorbate-2-phosphate, 10 mM/L of β-glycerophosphate) to induce osteoblast differentiation^71^. The medium was changed every 2–3 days, and the cells were differentiated for 21 days, after which differentiated cells were harvested for quantitative reverse transcription polymerase chain reaction (qRT-PCR).

### Alizarin red S staining

After being transfected with minicircle, osteoblasts were differentiated for 21 days. Following this, they were washed three times with PBS and fixed with 4% paraformaldehyde for 15 min at room temperature. The fixed cells were washed three times with deionized water (DW) and then incubated with 40 mM Alizarin Red S (ARS, ScienCell, Carlsbad, CA, USA) for 20 min at room temperature with gentle shaking. The stained cells were washed five times with DW and observed using an inverted routine microscope (Nikon Eclipse Ts2, Nikon, Tokyo, Japan).

### Animal care and OVX model

This animal study complied with the ARRIVE 2.0 guidelines. All surgical interventions, including presurgical and postsurgical animal care, were performed under the Laboratory Animals Welfare Act, the Guide for the Care and Use of Laboratory Animals, and the Guidelines and Policies for Rodent Survival Surgery provided by the IACUC (Institutional Animal Care and Use Committee) at the School of Medicine, Catholic University of Korea (Approval number: CUMS-2021-0259-07). Female Sprague–Dawley (SD) rats, 8-week-old and body weights of 200–220 g, were purchased from Orient Bio, Inc. (Seongnam, Korea). After 1 week quarantine and adaptation period, the rats were bred for 3 weeks and used for experiments at 12-weeks of age. SD rats were housed at a temperature of 20–26 °C, humidity of 50 ± 10%, and a 12-h light–dark cycle. Food was provided as a gamma lay sterilized diet (Altromin Spezialfutter GmbH & Co. KG, Germany), and water was provided as autoclaved R/O water.

To confirm the effect of the minicircles on bone formation, OVX was performed on 18 (12-week-old) female SD rats to induce postmenopausal osteoporosis. SD rats were anesthetized by isoflurane via inhalation system and the target area was shaved, sterilized, and disinfected. To expose the ovaries, two dorsolateral incisions were made, and the ovaries were removed. The muscle and skin layers were sutured and disinfected. During anesthesia, Duratears Ophtalmic Ointment (Alcon Korea Ltd.'s, Seoul, Korea) was applied to the rats to prevent their corneas from drying out. In addition, their condition was monitored until they regained consciousness after surgery. After OVX, the rats were administered gentamicin (intramuscularly 5 mg/kg) and ketoprofen (intramuscularly 5 mg/kg) for 3–7 days.

### Group allocation and minicircle injection

To confirm the expression of minicircles in normal SD rats, we intravenously injected mcOPG (80 μg/kg) or mcPTHrP (80 μg/kg) into 12-week-old female Sprague–Dawley rats (a total of 12 SD rats). The experimental groups were as follows: (1) the mcOPG group (n = 6), (2) mcPTHrP group (n = 6). We sacrificed two rats from each group on days 3, 7, and 15 after injection to analyze the protein expression of OPG and PTHrP in the kidney, spleen, and liver.

To confirm the pro-osteogenic effect of minicircles in OVX rats, the following procedures were performed on 24 SD rats randomly assigned 8 weeks after OVX induction and divided into six groups based on body weight (4 per group). One animal was randomly selected from each of the six cages, transferred to a new cage, and assigned a permanent number. The cages were then randomly assigned to each experimental group. This study investigates the effects of different treatments on a group of animals. The animals were divided into four experimental groups: (1) OVX group (n = 6), (2) OVX + mcOPG group (n = 6), (3) OVX + mcPTHrP group (n = 6), and (4) OVX + mcOPG + mcPTHrP (n = 6). The animals were randomly allocated to the groups by a third party. The veterinarians and breeders responsible for the care and treatment of the animals were not aware of the assigned group during the experiment.

After 8 weeks of ovariectomy (OVX), the rats were injected with mcOPG (80 µg/kg) through the tail vein. Similarly, after 16 weeks of OVX, mcPTHrP (80 µg/kg) was injected once a week for three weeks through the tail vein. The rats were sacrificed for histological and biochemical analysis after 24 weeks of OVX. The analysis excluded cases of euthanasia during the experiment and damage during sample collection after the animal experiment to humanely end the experiment. Therefore, the analysis was conducted on the following number of rats: (1) OVX group (n = 3), (2) OVX + mcOPG group (n = 4), (3) OVX + mcPTHrP group (n = 5), and (4) OVX + mcOPG + mcPTHrP group (n = 6).

### Polymerase chain reaction (PCR)

Transfected cells were harvested and incubated with TRIzol reagent (Thermo Fisher Scientific, Waltham, MA, USA) to extract the mRNA. The kidneys, livers, and spleens of rats were collected at 3, 7, and 15 days after minicircle injection, homogenized using a homogenizer (Bio-Gen PRO 200 homogenizer, PRO Scientific, Oxford, Connecticut, USA) with TRIzol, and mRNA was extracted. cDNA was synthesized from extracted RNA using a RevertAid First Strand cDNA Synthesis Kit (Thermo Fisher Scientific, Waltham, MA, USA), and the target genes were amplified using a PCR system. Gene expression was normalized to that of GAPDH, and quantification was performed using image J software. The primers used for PCR are listed in Supplementary Table 1.

### Quantitative reverse transcription polymerase chain reaction (qRT-PCR)

After 21 days of differentiation, the transfected hiPSC-derived osteoblasts were harvested, and cDNA was synthesized. qRT-PCR gene expression analyses were conducted on Light Cycler 480 (Roche Diagnostics, Indianapolis, USA) using LightCycler^®^ 480 SYBR Green 1 Master (Roche Diagnostics, Indianapolis, USA), according to the manufacturer’s instructions. The expression levels of target genes were normalized to that GAPDH, and the relative expression was calculated using the ΔΔCt method. The OPG/RANKL ratio was determined using the Ct method. (The primers used for qRT-PCR are listed in Supplementary Table 2).

### Western blotting analysis

Cells were harvested and lysed using radioimmunoassay precipitation buffer (RIPA, Sigma Aldrich, St. Louis, MO, USA) under constant shaking at 4 °C for 1 h, followed by centrifugation at 12,000 rpm for 20 min at 4 °C. For tissue samples, bones were harvested 24-weeks after OVX. Bones were homogenized and transferred into tissue protein extraction reagent (T-PER, Thermo Fisher Scientific, Waltham, MA, USA) supplemented with 1 protease inhibitor cocktail tablet (Roche Diagnostics, Indianapolis, IN, USA) and 1 mM of PMSF under constant shaking at 4 °C for 2 h. The extracted protein samples were quantified using Bicinchoninic Acid (BCA, Thermo Fisher Scientific, Waltham, MA, USA) protein assay. Electrophoretic separation of proteins was performed using sodium dodecyl sulfate (SDS)-sulfate–polyacrylamide gels and transferred onto nitrocellulose blotting membranes (GE Healthcare, Chicago, Illinois, USA). Membranes were washed with Tris-buffered saline supplemented with Tween-20 (TBST) and blocked with 3% bovine serum albumin (BSA; Sigma Aldrich, St. Louis, MO, USA) or 3% non-fat milk (BD Difco, NJ, USA) with TBST. The membranes were incubated with specific primary antibodies (Supplementary Table 3) overnight at 4 °C. After washing, the membranes were incubated with the appropriate secondary antibodies. Protein expression was assessed using ECL solution (AbFrontier, Seoul, South Korea), followed by exposure of the membrane to the ImageQuant LAS 4000 system (BioRad, Hercules, CA, USA). Quantification of protein band intensity was performed using the ImageJ software. Band intensity of target proteins was normalized to that of GAPDH or β-Actin.

### Micro-CT analysis

After 24 weeks of OVX, four groups of rats were sacrificed: the OVX group (n = 3), the OVX + mcOPG group (n = 4), the OVX + mcPTHrP group (n = 5), and the OVX + mcOPG + mcPTHrP group (n = 6). Femur samples were collected for micro-CT analysis using a SkyScan 1173 µCT scanner (Bruker, Billerica, MA, USA) with a source voltage of 130 kV and source current of 60 µA, and a voxel size of 12.42 mm^3^. The volume of interest (VOI) for trabecular bone analysis in the proximal femur was selected to be 5.5 mm thick, beginning below the femoral head and including the primary spongiosa in the greater trochanter region. This VOI was communicated to the operators at OBEN, Inc. (Suwon, Korea) who conducted the micro-CT scan. The trabecular bone microstructure of the ROI was evaluated using various parameters, including BMD, BV/TV, trabecular number (Tb.N), trabecular thickness (Tb.Th), BS, TV, BV, specific bone surface (BS/BV), bone surface density (BS/TV), trabecular separation (Tb.Sp), DA, connectivity density (Conn.D), and SMI, in accordance with the guidelines for evaluating bone microstructure in rodents^72^. The images were reconstructed using SkyScan NRecon reconstruction software (version 1.7.4.6).

### Immunofluorescence staining

Detection minicircle DNA vector distribution in vivo was performed using 15 female SD rats (12-week-old). mcOPG (80 µg/kg) and mcPTHrP (80 µg/kg) were delivered via hydrodynamic intravenous injection into the tail vein. At 3, 7, and 15 days after injection, the rats were sacrificed, and tissue samples of the spleen, kidney, and liver were collected for histological and biochemical analyses. Kidney, spleen, and liver samples were fixed with 4% PFA for 24 h and incubated in 15 and 30% sucrose for 24 h, respectively. Samples were embedded using optimal cutting temperature compound (OCT, Sakura Finetek, Torrance, CA, USA) and cut into 5 μm thick section using a cryo-microtome (Thermo Fisher Scientific, Waltham, MA, USA). Cryo-sectioned slides were placed in cold acetone for 10 min and incubated in citrated buffer (Sigma Aldrich, St. Louis, MO, USA) for 20 min at 60 °C for antigen retrieval. The slides were permeabilized using 0.1% Triton X-100 in PBS and blocked with PBS containing 10% normal goat serum and 0.1% Triton X-100 for 1 h at RT. The slides were incubated overnight at 4 °C with primary antibodies diluted in the blocking solution (1:50). After washing, the slides were incubated with secondary antibodies, followed by nuclear staining with 4',6-Diamidine-2'-phenylindole dihydrochloride (DAPI). Subsequently, the slides were mounted with an antifade mounting reagent (Thermo Fisher Scientific, Waltham, MA, USA) after blocking autofluorescence. The stained slides were observed under an upright fluorescence microscope (Axio Imager. M2, Carl Zeiss, Oberkochen, Germany).

To confirm the osteogenic effect of the minicircles on bone in the OVX rat model, rat femur samples were fixed in 4% PFA for 24 h. The samples were rinsed in running tap water for 24 h and incubated with a decalcifying solution (Sigma Aldrich, St. Louis, MO, USA) for 1 month at 4 °C, and the solution was changed once every 2 days. Easy puncture of the bones using a needle confirmed successful demineralization. After decalcification, samples were embedded in paraffin (Leica Biosystems, Nußloch, Baden-Wurttemberg, Germany) and sectioned using a microtome (Thermo Fisher Scientific, Waltham, MA, USA). Sectioned slides were placed in a 60 °C oven and deparaffinized using xylene for 15 min and then rehydrated in a sequentially decreasing ethanol series. The slides were permeabilized using 3% hydrogen peroxide (H_2_O_2_) and washed with cold tap water, followed by blocking with 1 × TBS containing 10% normal goat serum. The slides were incubated overnight at 4 °C with primary antibodies (Supplementary Table 3) diluted in the blocking solution (1:200). After washing and incubating with the appropriate secondary antibodies, the slides were counterstained with DAPI. Subsequently, autofluorescence was blocked using Vector TrueVIEW Autofluorescence Quenching Kit (VECTOR Laboratories, Newark, CA, USA), following the manufacturer’s instructions. Slides were mounted using an antifade mounting reagent (Thermo Fisher Scientific, Waltham, MA, USA), and observed under an upright fluorescence microscope (Axio Imager. M2, Carl Zeiss, Oberkochen, Germany).

### Hemoxylin and eosin (H&E) staining

Briefly, tissue samples were deparaffinized, rehydrated, and the slides were stained with Mayer’s hematoxylin (Sigma Aldrich, St. Louis, MO, USA) solution for 15 min, dipped in cold tap water and 1% acid ethanol (1% HCl in 70% ethanol), and rinsed in tap water. The slides were immersed in 0.2% ammonia water and stained with eosin. After dehydration, the slides were mounted using a VectaMount permanent mounting medium (VECTOR Laboratories, Newark, CA, USA).

### Statistical analysis

The experiments were conducted at least three times, and the data obtained are continuous scales. The data included four or more independent groups (independent variables), and each group was observed independently. Normality was checked using the Shapiro–Wilk test, and spurious outliers were identified. The data were analyzed using a one-way ANOVA after confirming equal variance through Levene's test. Post hoc analyses were conducted using Fisher's least significant difference (LSD). When equal variance was not satisfied, Welch's ANOVA test was performed, and comparisons between groups were analyzed using t-tests. Statistical analyses were conducted using SPSS (IBM Corporation, Armonk, NY, USA), and graphs were created using GraphPad Prism 9 (GraphPad Software Inc., San Diego, CA, USA). Statistical significance was set at *P* < 0.05 (*,# *P* < 0.05, **,## *P* < 0.01, ***,### *P* < 0.001, and n. s. = not significant).

### Supplementary Information

Supplementary Information.

## Data Availability

The datasets generated during and/or analyzed during the current study are available from the corresponding author on reasonable request.

## References

[1] Sandhu SK, Hampson G (2011). The pathogenesis, diagnosis, investigation and management of osteoporosis. J. Clin. Pathol..

[2] Dobbs MB, Buckwalter J, Saltzman C (1999). Osteoporosis: The increasing role of the orthopaedist. Iowa Orthop. J..

[3] Mirza F, Canalis E (2015). Management of endocrine disease: Secondary osteoporosis: Pathophysiology and management. Eur. J. Endocrinol..

[4] NIH Consensus Development Panel on Osteoporosis Prevention, Diagnosis, and Therapy (2001). Osteoporosis prevention, diagnosis, and therapy. JAMA.

[5] Chen X (2018). Osteoblast-osteoclast interactions. Connect. Tissue Res..

[6] Liang B, Burley G, Lin S, Shi YC (2022). Osteoporosis pathogenesis and treatment: Existing and emerging avenues. Cell Mol. Biol. Lett..

[7] Tobeiha M, Moghadasian MH, Amin N, Jafarnejad S (2020). RANKL/RANK/OPG pathway: A mechanism involved in exercise-induced bone remodeling. Biomed. Res. Int..

[8] Ji MX, Yu Q (2015). Primary osteoporosis in postmenopausal women. Chronic Dis. Transl. Med..

[9] Eastell R (2016). Postmenopausal osteoporosis. Nat. Rev. Dis. Primers.

[10] Eastell R (2019). Pharmacological management of osteoporosis in postmenopausal women: An endocrine society* clinical practice guideline. J. Clin. Endocrinol. Metab..

[11] Arceo-Mendoza RM, Camacho PM (2021). Postmenopausal osteoporosis: Latest guidelines. Endocrinol. Metab. Clin. North Am..

[12] Brent MB (2023). Pharmaceutical treatment of bone loss: From animal models and drug development to future treatment strategies. Pharmacol. Ther..

[13] Chen JS, Sambrook PN (2011). Antiresorptive therapies for osteoporosis: A clinical overview. Nat. Rev. Endocrinol..

[14] Camacho PM (2020). American Association of Clinical Endocrinologists/American College of Endocrinology Clinical Practice guidelines for the diagnosis and treatment of postmenopausal osteoporosis-2020 update. Endocr. Pract..

[15] Rosen CJ (2005). Clinical practice. Postmenopausal osteoporosis. N. Engl. J. Med..

[16] LeBoff MS (2022). The clinician's guide to prevention and treatment of osteoporosis. Osteoporos. Int..

[17] Brown JP (2021). Long-term treatment of postmenopausal osteoporosis. Endocrinol. Metab. (Seoul).

[18] Gregson CL (2022). UK clinical guideline for the prevention and treatment of osteoporosis. Arch. Osteoporos..

[19] Anagnostis P (2019). New therapeutic targets for osteoporosis. Maturitas.

[20] Cosman F (2014). Combination therapy for osteoporosis: A reappraisal. Bonekey Rep..

[21] Zhang C, Song C (2020). Combination therapy of PTH and antiresorptive drugs on osteoporosis: A review of treatment alternatives. Front. Pharmacol..

[22] Leder BZ (2015). Effects of abaloparatide, a human parathyroid hormone-related peptide analog, on bone mineral density in postmenopausal women with osteoporosis. J. Clin. Endocrinol. Metab..

[23] Neer RM (2001). Effect of parathyroid hormone (1–34) on fractures and bone mineral density in postmenopausal women with osteoporosis. N. Engl. J. Med..

[24] Nakajima M (2000). Effect of intermittent administration of human parathyroid hormone (1–34) on the mandibular condyle of ovariectomized rats. J. Bone Miner. Metab..

[25] Osagie-Clouard L (2017). Parathyroid hormone 1–34 and skeletal anabolic action: The use of parathyroid hormone in bone formation. Bone Jt. Res..

[26] Hong P (2023). Is abaloparatide more efficacious on increasing bone mineral density than teriparatide for women with postmenopausal osteoporosis? An updated meta-analysis. J. Orthop. Surg. Res..

[27] Sleeman A, Clements JN (2019). Abaloparatide: A new pharmacological option for osteoporosis. Am. J. Health Syst. Pharm..

[28] Hattersley G, Dean T, Corbin BA, Bahar H, Gardella TJ (2016). Binding selectivity of abaloparatide for PTH-Type-1-receptor conformations and effects on downstream signaling. Endocrinology.

[29] Brent MB (2021). Abaloparatide: A review of preclinical and clinical studies. Eur. J. Pharmacol..

[30] Kim JW (2021). Increased potential of bone formation with the intravenous injection of a parathyroid hormone-related protein minicircle DNA Vector. Int. J. Mol. Sci..

[31] Boyce BF, Xing L (2007). Biology of RANK, RANKL, and osteoprotegerin. Arthritis Res. Ther..

[32] Wada T, Nakashima T, Hiroshi N, Penninger JM (2006). RANKL-RANK signaling in osteoclastogenesis and bone disease. Trends Mol. Med..

[33] Hofbauer LC, Schoppet M (2004). Clinical implications of the osteoprotegerin/RANKL/RANK system for bone and vascular diseases. JAMA.

[34] Doolittle ML (2020). Genetic analysis of osteoblast activity identifies Zbtb40 as a regulator of osteoblast activity and bone mass. PLoS Genet..

[35] Gentleman E (2009). Comparative materials differences revealed in engineered bone as a function of cell-specific differentiation. Nat. Mater..

[36] Bernar A, Gebetsberger JV, Bauer M, Streif W, Schirmer M (2022). Optimization of the alizarin red S assay by enhancing mineralization of osteoblasts. Int. J. Mol. Sci..

[37] Rutkovskiy A, Stenslokken KO, Vaage IJ (2016). Osteoblast differentiation at a glance. Med. Sci. Monit. Basic Res..

[38] Wilson SR, Peters C, Saftig P, Bromme D (2009). Cathepsin K activity-dependent regulation of osteoclast actin ring formation and bone resorption. J. Biol. Chem..

[39] Costa AG, Cusano NE, Silva BC, Cremers S, Bilezikian JP (2011). Cathepsin K: Its skeletal actions and role as a therapeutic target in osteoporosis. Nat. Rev. Rheumatol..

[40] Solomon DH, Rekedal L, Cadarette SM (2009). Osteoporosis treatments and adverse events. Curr. Opin. Rheumatol..

[41] Tonk CH (2022). Therapeutic treatments for osteoporosis-which combination of pills is the best among the bad?. Int. J. Mol. Sci..

[42] Wei K, Qu Y, Gao Y, Ma Y (2021). Comparison of efficacy of teriparatide (parathyroid hormone 1–34) alone and in combination with zoledronic acid for osteoporosis in postmenopausal women. J. Coll. Physicians Surg. Pak..

[43] Huang M (2009). Novel minicircle vector for gene therapy in murine myocardial infarction. Circulation.

[44] Kay MA, He CY, Chen ZY (2010). A robust system for production of minicircle DNA vectors. Nat. Biotechnol..

[45] Gill DR, Pringle IA, Hyde SC (2009). Progress and prospects: The design and production of plasmid vectors. Gene Ther..

[46] Sendra L, Herrero MJ, Alino SF (2018). Translational advances of hydrofection by hydrodynamic injection. Genes (Basel).

[47] Rim YA (2020). Chondrogenic differentiation from induced pluripotent stem cells using non-viral minicircle vectors. Cells.

[48] Park N (2017). Etanercept-synthesising mesenchymal stem cells efficiently ameliorate collagen-induced arthritis. Sci. Rep..

[49] Yi H (2014). A new strategy to deliver synthetic protein drugs: Self-reproducible biologics using minicircles. Sci. Rep..

[50] Rim YA (2014). Self in vivo production of a synthetic biological drug CTLA4Ig using a minicircle vector. Sci. Rep..

[51] Datta NS, Abou-Samra AB (2009). PTH and PTHrP signaling in osteoblasts. Cell. Signal..

[52] Manolagas SC (2000). Birth and death of bone cells: Basic regulatory mechanisms and implications for the pathogenesis and treatment of osteoporosis. Endocr. Rev..

[53] Baron R, Ferrari S, Russell RG (2011). Denosumab and bisphosphonates: Different mechanisms of action and effects. Bone.

[54] Yu H, de Vos P, Ren Y (2011). Overexpression of osteoprotegerin promotes preosteoblast differentiation to mature osteoblasts. Angle Orthod..

[55] Wang YH, Liu Y, Maye P, Rowe DW (2006). Examination of mineralized nodule formation in living osteoblastic cultures using fluorescent dyes. Biotechnol. Prog..

[56] Palumbo S, Li WJ (2013). Osteoprotegerin enhances osteogenesis of human mesenchymal stem cells. Tissue Eng. A.

[57] Yao Y (2011). Synergistic enhancement of new bone formation by recombinant human bone morphogenetic protein-2 and osteoprotegerin in trans-sutural distraction osteogenesis: a pilot study in dogs. J. Oral Maxillofac. Surg..

[58] Turner RT (2001). Animal models for osteoporosis. Rev. Endocr. Metab. Disord..

[59] Hadjidakis DJ, Androulakis II (2006). Bone remodeling. Ann. N. Y. Acad. Sci..

[60] Riggs BL, Parfitt AM (2005). Drugs used to treat osteoporosis: the critical need for a uniform nomenclature based on their action on bone remodeling. J. Bone Miner. Res..

[61] Khosla S, Hofbauer LC (2017). Osteoporosis treatment: Recent developments and ongoing challenges. Lancet Diabetes Endocrinol..

[62] Finkelstein JS, Wyland JJ, Lee H, Neer RM (2010). Effects of teriparatide, alendronate, or both in women with postmenopausal osteoporosis. J. Clin. Endocrinol. Metab..

[63] Cosman F (2011). Effects of intravenous zoledronic acid plus subcutaneous teriparatide [rhPTH(1–34)] in postmenopausal osteoporosis. J. Bone Miner. Res..

[64] Tsai JN (2013). Teriparatide and denosumab, alone or combined, in women with postmenopausal osteoporosis: The DATA study randomised trial. Lancet.

[65] Leder BZ (2015). Denosumab and teriparatide transitions in postmenopausal osteoporosis (the DATA-Switch study): Extension of a randomised controlled trial. Lancet.

[66] Idolazzi L (2016). Teriparatide and denosumab combination therapy and skeletal metabolism. Osteoporos. Int..

[67] Nakamura Y (2017). Two-year clinical outcome of denosumab treatment alone and in combination with teriparatide in Japanese treatment-naive postmenopausal osteoporotic women. Bone Res..

[68] Suzuki T, Nakamura Y, Kato H (2019). Efficacy of 4-year denosumab treatment alone or in combination with teriparatide in Japanese postmenopausal osteoporotic women. Mod. Rheumatol..

[69] Sun Y (2022). Efficacy of the combination of teriparatide and denosumab in the treatment of postmenopausal osteoporosis: A meta-analysis. Front. Pharmacol..

[70] Rim YA (2018). Recent progress of national banking project on homozygous HLA-typed induced pluripotent stem cells in South Korea. J. Tissue Eng. Regen. Med..

[71] Jung H, Rim YA, Park N, Nam Y, Ju JH (2021). Restoration of osteogenesis by CRISPR/Cas9 genome editing of the mutated COL1A1 gene in osteogenesis imperfecta. J. Clin. Med..

[72] Bouxsein ML (2010). Guidelines for assessment of bone microstructure in rodents using micro-computed tomography. J. Bone Miner. Res..

