# Supplementary material for: Effects of stepwise administration of osteoprotegerin and parathyroid hormone-related peptide DNA vectors on bone formation in ovariectomized rat model
Source: Sci Rep. 2024 Jan 30;14:2477. doi: 10.1038/s41598-024-51957-0 (PMC10827729; doi:10.1038/s41598-024-51957-0)
Supplement: Supplementary file 1 — Supplementary Information. [file 41598_2024_51957_MOESM1_ESM.pdf]

Supplementary Information for

Effects of Stepwise Administration of Osteoprotegerin and Parathyroid hormone-related peptide DNA Vectors on Bone Formation in Ovariectomized Rat Model

Ye Ji Eom[1,2], Jang-Woon Kim[1], Yeri Alice Rim[1,*], Jooyoung Lim[1,2], Se In Jung[1,2], and Ji Hyeon Ju[1,3,*]

[1]Catholic iPSC Research Center (CiRC), CiSTEM Laboratory, College of Medicine, The Catholic University of Korea, Seoul, Republic of Korea;

[2] Department of Biomedicine and Health Science, College of Medicine, The Catholic University of Korea, Seoul, Republic of Korea;

[3]Division of Rheumatology, Department of Internal Medicine, Seoul St. Mary's Hospital, Institute of Medical Science, College of Medicine, The Catholic University of Korea, Seoul, Republic of Korea;

*Correspondence:
llyerill0114@gmail.com (Yeri Alice Rim); Tel.: +82-2-3147-8936
juji@catholic.ac.kr (Ji Hyeon Ju); Tel.: +82-2-3147-8825; Fax: +82-2-3476-2274

Supplemental Table 1 : Primer sequences for PCR experiments.

**Gene Names**	**Species**	**Primers**		**Product size(bp)**
OPG	Human	Forward	5'-CGCTACCTTGAGATAGAGTT-3'	340
		Reverse	5'-CCAAGACACTAAGCCAGTTA-3'	
PTHrP	Human	Forward	5'-CARGACAAGGGGAAGTCCAT-3'	158
		Reverse	5'-GACGTTGTGGAGGTGTCAGA-3'	
GAPDH	Rat	Forward	5'-CAGGGCTGCCTTCTCTTGTG-3'	169
		Reverse	5'-GGTGATGGGTTTCCCGTTGA-3'	

Supplemental Table 2 : Primer sequences for qRT-PCR experiments.

**Gene Names**	**Species**	**Primers**		**Product size(bp)**
OCT4	Human	Forward	5’-ACCCCTGGTGCCGTGAA-3’	190
		Reverse	5’-GGCTGAATACCTTCCCAAATA-3’	
OPG	Human	Forward	5’-GGACATGCTAACCTCACCT-3’	213
		Reverse	5’-GTGGTACGTCTTTGAGTGCT-3’	
PTHrP	Human	Forward	5’-CARGACAAGGGGAAGTCCAT-3’	158
		Reverse	5’-GACGTTGTGGAGGTGTCAGA-3’	
COL1A1	Human	Forward	5’-TCTGCGACAACGGCAAGGTG-3’	146
		Reverse	5’-GACGCCGGTGGTTTCTTGGT-3’	
OCN	Human	Forward	5’-CGCTACCTGTATCAATGGCTGG-3’	123
		Reverse	5’-CTCCTGAAAGCCGATGTGGTCA-3’	
RANKL	Human	Forward	5’-ACATATCGTTGGATCACAGCACAT-3’	100
		Reverse	5’-CAAAGGCTGAGCTTCAAGCT-3’	
GAPDH	Human	Forward	5’-CTGTTGCTGTAGCCAAATTCGT-3’	101
		Reverse	5’-ACCCACTCCTCCACCTTTGA-3’	

Supplemental Table 3 : Antibodies

**Product name**	**Catalog #**	**Host species**	**Application**	**Dilution**	**Application specific details(e.g. antigen retrieval, blocking, incubation)**
OPG antibody (E-10)	Santa Cruz, sc-390518	Mouse	WB	1:500	3% Skim milk in TBS-0.1% Tween, overnight, 4°C
			IFA	1:50	10% Normal Goat Serum in PBS - 0.1% Triton X-100, overnight, 4°C
PTHLP/PTHrP Antibody	Novusbio, NBP2-94646	Rabbit	WB	1:500	3% Skim milk in TBS-0.1% Tween, overnight, 4°C
			IFA	1:50	10% Normal Goat Serum in PBS - 0.1% Triton X-100, overnight, 4°C
COL1A1 (C-18)	Santa Cruz, sc-8784	Goat, Rabbit	WB	1:500	3% BSA in TBS-0.1% Tween, overnight, 4°C
			IFA	1:200	10% Normal Human Serum in TBS, overnight, 4°C
Osteocalcin (G-5)	Santa Cruz, sc-365797	Mouse	IFA	1:200	10% Normal Goat Serum in TBS, overnight, 4°C
RANKL (G-1) antibody	Santa Cruz, sc-377079	Mouse	WB	1:500	3% BSA in TBS-0.1% Tween, overnight, 4°C
			IFA	1:200	10% Normal Goat Serum in TBS, overnight, 4°C
Anti-RUNX2 antibody	Abcam, ab76956	Mouse	WB	1:500	3% BSA in TBS-0.1% Tween, overnight, 4°C
			IFA	1:200	10% Normal Goat Serum in TBS, overnight, 4°C
Cathepsin K antibody (E-7)	Santa Cruz, sc-48353	Mouse	IFA	1:200	10% Normal Goat Serum in TBS, overnight, 4°C
Anti-GAPDH antibody [6C5]	Abcam, ab8245	Mouse	WB	1:5000	3% BSA in TBS-0.1% Tween, overnight, 4°C
Anti-beta Actin antibody	Abcam, ab227387	Rabbit	WB	1:5000	3% BSA in TBS-0.1% Tween, overnight, 4°C
Alexa Fluor 594 goat anti-mouse IgG (H+L)	Invitrogen, A11032	Goat	IFA	1:200	10% Normal Goat Serum in PBS - 0.1% Triton X-100, 1hr, RT
				1:500	TBS-0.1% Tween, 1hr, RT
Alexa Fluor 488 goat anti-mouse IgG (H+L)	Invitrogen, A11029	Goat	IFA	1:200	10% Normal Goat Serum in PBS - 0.1% Triton X-100, 1hr, RT
				1:500	TBS-0.1% Tween, 1hr, RT

Supplementary Figure 1. : Unprocessed RT-PCR electrophoresis gels and western blots. Source data for Figure 1f and 1g.

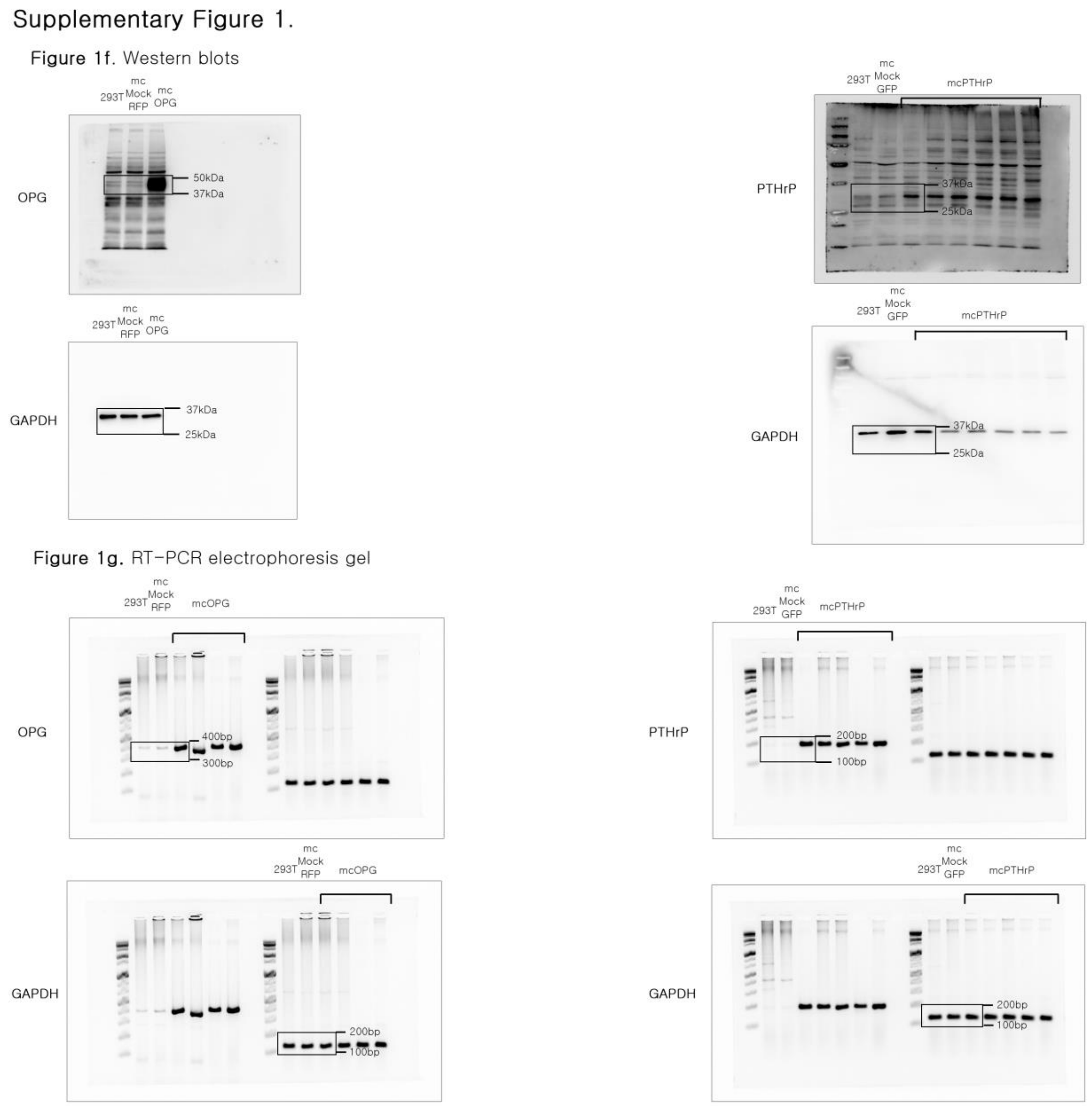

Supplementary Figure 2. : Unprocessed RT-PCR electrophoresis gels. Source data for Figure 3e, 3f, and 3g.

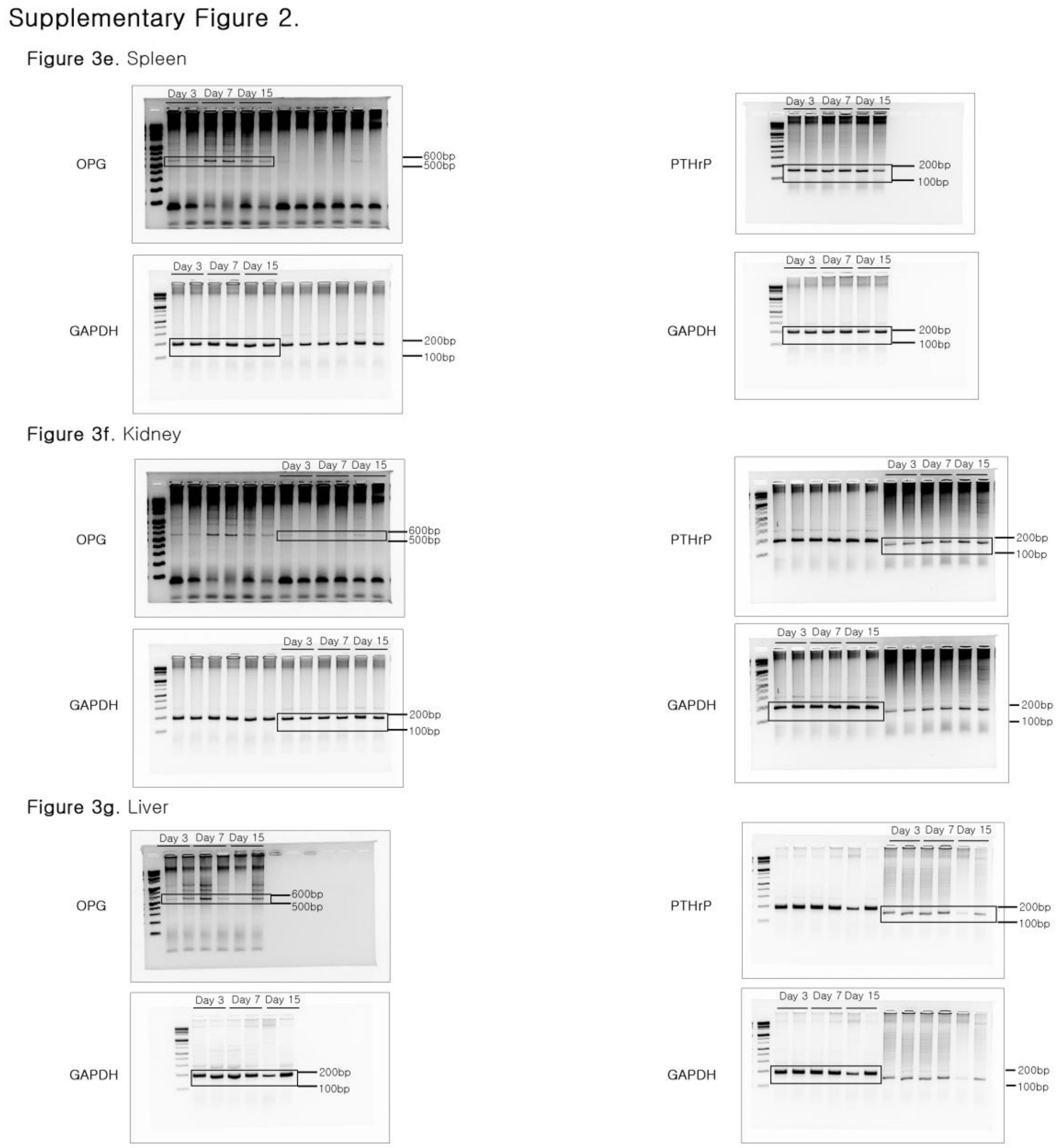

Supplementary Figure 3. : Unprocessed western blots. Source data for Figure 5c.

Supplementary Figure 3.

Figure 5c. Western blots

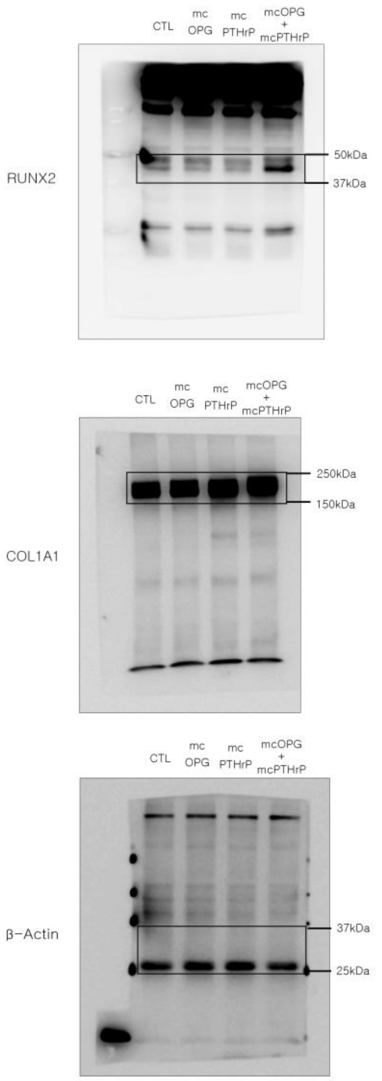

Supplementary Figure 4. : Unprocessed western blots. Source data for Figure 6c.

Supplementary Figure 4.

Figure 6c. Western blots

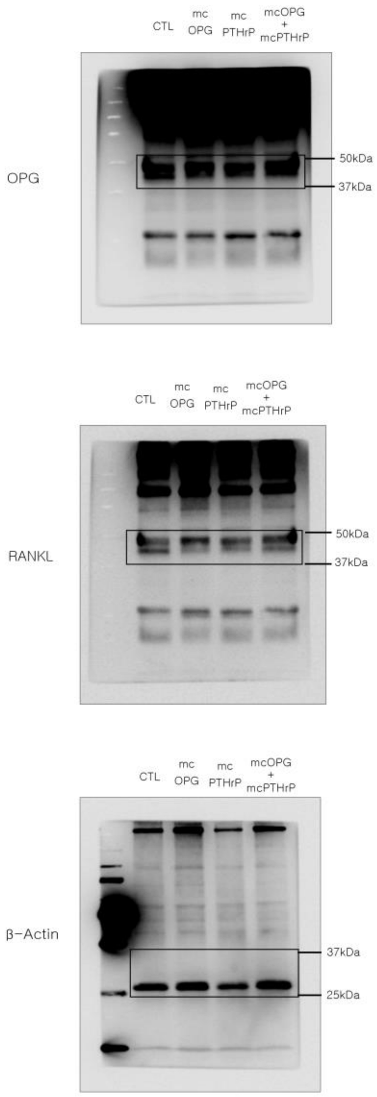

Supplementary Figure 5. micro-CT parameters and hematoxylin and eosin (H&E) staining images.

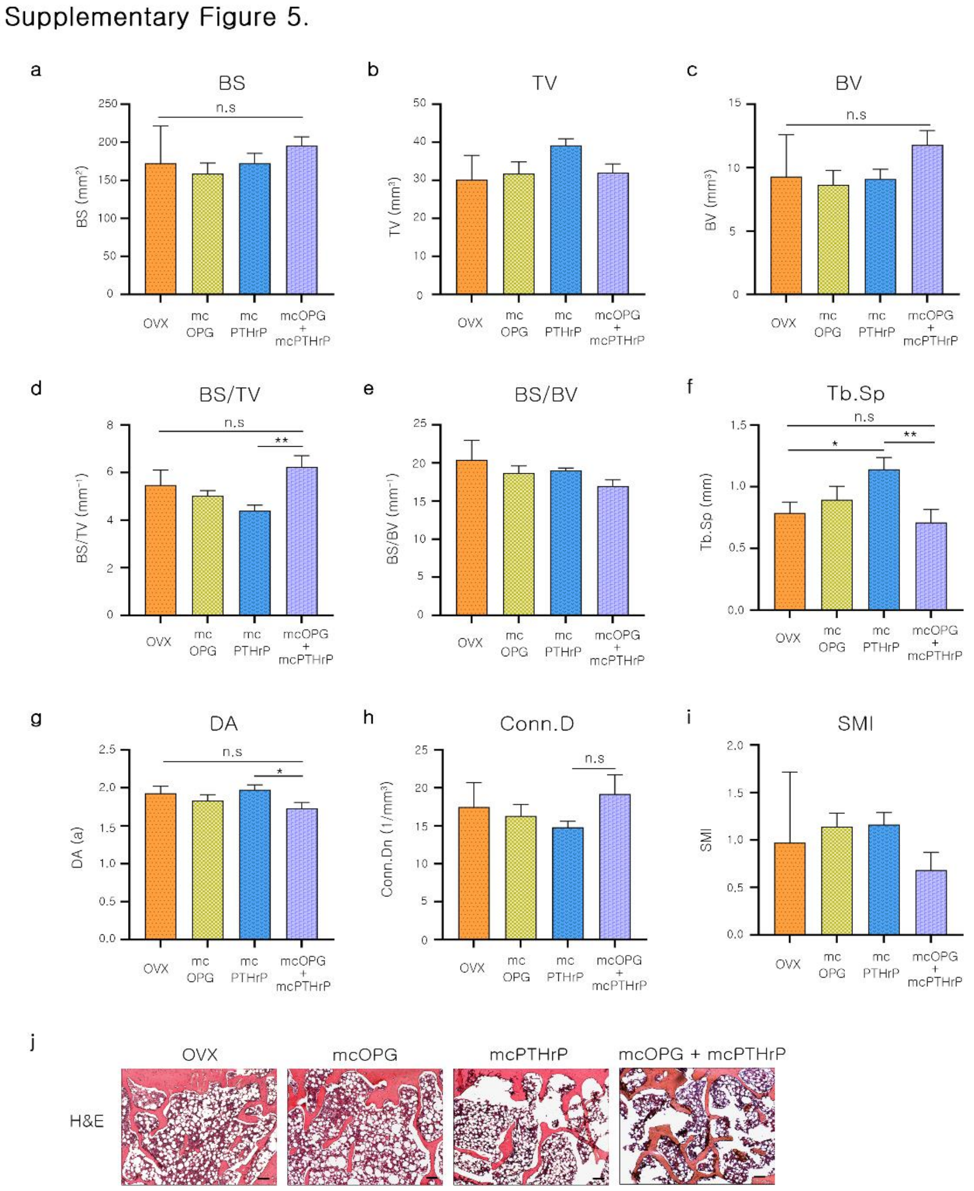

Supplementary Figure 5. Micro-CT parameters and hematoxylin and eosin (H&E) staining images. (a-j) Micro-CT scan was performed to assess bone-related parameters in the femur of OVX rats injected with minicircles encoding OPG and PTHrP. (a) Bone surface (BS), (B) total volume (TV), (c) bone volume (BV), (d) specific bone surface (BS/BV), (e) bone surface density (BS/TV), and (f) trabecular separation. (Tb.Sp), (g) degree of anisotropy (DA), and (h) connectivity density (Conn.D), and (i) structure model index (SMI). Data are presented as mean ± SEM. One-way ANOVA; *P < 0.05; **P < 0.01; ***P < 0.001; n. s. = not significant, LSD test. (j) Hematoxylin and eosin (H&E) staining images of rat femurs from each group. Scale bar = 100μm. OVX: ovariectomy; mcOPG: minicircle encoding osteoprotegerin; mcPTHrP: minicircle encoding parathyroid hormone related-protein; OPG: osteoprotegerin; PTHrP: parathyroid hormone related protein; H&E: hematoxylin and eosin.
